# The Author Truncation “et al.” in Article References: An Anachronism That Needs to Change

**DOI:** 10.1177/22925503211051109

**Published:** 2021-11-17

**Authors:** Achilles Thoma, Jessica Murphy, Charlie H. Goldsmith

**Affiliations:** 1 3710 Department of Surgery, Division of Plastic Surgery, McMaster University, Hamilton, ON, Canada; 2 Department of Health Research Methods, Evidence and Impact (HEI), Health Sciences, 3710 McMaster University, Hamilton, ON, Canada

**Keywords:** auteurs, publications de chirurgie plastiquet, reconnaissance, et coll

## Abstract

**Background:** Valuable research requires contribution from many experts; however, the “et al.” truncation often keeps all individuals from being acknowledged. The adoption of a new citation rule (*list all authors up to 30, followed by et al.*) would allow more authors to be acknowledged. The purpose of this study was to (1) explore the citation styles of the top 10 Plastic Surgery, Surgery, and Medical journals and (2) compare the number of extra pages required, and the number of additional authors acknowledged when the “new rule” is implemented. **Methods:** The top 10 journals in Plastic Surgery, Surgery, and Medicine were identified. The citation styles used in each of the journals were reviewed and the reference list from a recently published article was extracted. The original reference list was used to create an Extended Reference List using the new rule. **Results:** Most journals implemented “et al.” when seven or more authors were listed. Ten articles required additional pages to accommodate the Extended Reference List. When the “et al.” truncation was introduced after 30 authors, there was an almost 100% chance of all authors being included. The adoption of this rule rarely resulted in the need for additional pages, especially within Plastic Surgery. **Conclusions:** In a time of electronic publishing, where constraints such as article and journal page length should not be important factors, all authors should be recognized. The use of the “et al.” truncation should be discouraged by all individuals involved in the production and publication of research.

**Scenario**

You are asked by the Editor-in-Chief of your specialty's journal to review an article in your area of expertise. You gladly accept the task. One of the questions you are required to answer in your review is whether the authors of the submitted manuscript have missed any important articles in their references. As you are the recognized expert in this field, you glance at the references to see if a key article you published 3 years earlier has been included. The first author of that article was a junior resident in your service and the research was done under your supervision. To their credit, the authors included the said article, but you are dismayed that the reference does not include your name. It includes only the names of the first three authors, all junior residents in your service. Your name, and the names of many others, are lost in the et al. truncation.

## Résumé

**Contexte:** Une recherche intéressante nécessite la contribution de nombreux experts; cependant, le raccourci « et coll. » empêche souvent la reconnaissance de toutes les personnes. L'adoption d'une nouvelle règle de référencement (énumérer tous les auteurs jusqu'à 30, suivi de *et coll.*) permettrait de reconnaître davantage d'auteurs. L'objectif de cette étude était de 1) explorer les styles de référence des 10 principaux journaux de chirurgie plastique, chirurgie et médecine, et 2) comparer le nombre de pages supplémentaires requises et le nombre d'auteurs supplémentaires identifiés quand la « nouvelle règle » est appliquée. **Méthodes:** Les 10 principaux journaux de chirurgie plastique, chirurgie et médecine ont été identifiés. Les styles de référencement utilisés dans chacun de ces journaux ont été revus et la liste des références d'un article récemment publié a été extraite. La liste de références originale a servi à créer une liste de références étendue selon la nouvelle règle. **Résultats:** La majorité des journaux a utilisé « et coll. » quand 7 auteurs ou plus étaient cités. Dix articles ont nécessité des pages supplémentaires pour accueillir la liste de références étendue. Quand le raccourci « et coll. » était introduit avec le seuil de 30 auteurs, il y avait pratiquement 100 % de chances d'avoir inclus tous les auteurs. L'adoption de cette règle a rarement entraîné le besoin de pages supplémentaires, en particulier dans *Plastic Surgery*. **Conclusions:** À l'époque de l'édition électronique où des contraintes telles que la longueur d'un article et le nombre de pages ne devraient pas être des facteurs importants, tous les auteurs devraient être reconnus. L'utilisation du raccourci « et coll. » devrait être découragée par tous ceux qui sont impliqués dans la production et la publication de recherches.

## Introduction

In modern-day medicine, valuable published research is the product of many experts’ contributions. Such experts may include surgeons, research methodologists, biostatisticians, health economists, and medical librarians. It is imperative that each individual involved in published research is given the proper acknowledgment based on their contributions. One area of author acknowledgment that has received considerable attention is that of “author bloat” or “author inflation”. Over the past decades, there has been a noted increase in the number of listed authors in published literature across disciplines.^1–3^ This phenomenon is believed to be due to many potential factors such as increased collaboration, heightened research complexity, the belief that listing senior authors will facilitate publication, and honorary authorship.^2,4,5^ Honorary authorship is when a listed author did not meaningfully contribute to the paper, and could not defend their roles in the publication if needed.^5,6^ Often, such contributions include reviewing or approving a manuscript, recruiting study subjects, recruiting co-authors, supervising the project, or providing illustrations.^7^ Many researchers have speculated that research complexity cannot adequately explain “author bloat”, and many believe that the other factors, including honorary authorship can help explain this occurrence.^4,8,9^ The concept of author bloat and honorary authorship is a problem across disciplines and contributes to the issue discussed in this paper, the “et al.” truncation. As such, these concepts should be considered when reading this paper.

The “et al.” truncation is often placed after the third listed author in a citation within the “Reference” section of a manuscript. The issue with the use of “et al.” is that it “cuts-off” the names of any authors listed after the third author, thereby, not acknowledging individuals with legitimate contributions to scientific work as alluded in our scenario. Whether an author is affected by this truncation depends on the number of listed authors, the order of authorship, and the chosen citation style. Standards for author order vary considerably across disciplines;^10^ some use alphabetical order^11^ and others will randomize author order to give equal credit to all authors.^12,13^ Others will list the senior author in the last “slot” and the individual who contributed the most work in the first “slot”.^10^ In this common scenario, the senior author would be “cut” from a citation; however, this truncation is also particularly discriminatory to others. As an example, biostatisticians are an important group of experts in clinical research; however, they are often listed near the end of a long list of authors. A previous publication found that when the “et al.” was placed after the first three listed authors, the biostatistician's name appeared in 47% of citations. This increased to 80% when “et al.” was placed after the first six authors.^14^ Similar to author order, the use of the “et al.” truncation also differs across disciplines based on the citation style used (Table 1). The use of this truncation, however, also seems to be at the discretion of the publishing journal. In the days of “hard copy” publications, one can understand the use of the “et al.” truncation as it would save space, thereby saving on printing costs.

**Table 1.** Common Citation Styles.

Style	Discipline	Application
MLA	Humanities	In publications with 3 or more authors, list the first author followed by “et al.”
APA	Social and Behavioral Sciences	In publications with more than 20 authors, list the first 19 authors then use an ellipsis (…) followed by the final author's name
Chicago	History and Humanities	In publications with more than 10 authors, list the first 7 authors followed by “et al.”
AMA	Medical	In publications with more than 6 authors, list the first 3 authors followed by “et al.”
Vancouver	Medical and Sciences	In publications with more than 6 authors, list the first 6 authors followed by “et al.”

Note: Information was collected in Februar*y* 2020.

Abbreviations: APA, American Psychological Association; AMA, American Medical Association; MLA, Modern Language Association.

As journals, however, move increasingly to electronic-only publishing, the costs associated with physical space, printing, and shipping is moot. As such, proper recognition of authors is being denied without just cause. The primary purpose of this article is to explore the practice of using “et al.” within the Plastic Surgery literature references and compare this practice in the general surgery and medical literature. The secondary purpose is to estimate the extra page print burden that would occur if the “et al.” truncation was used after the first 30 authors. This is based on a previous publication by Goldsmith and colleagues,^14^ who found that if the “et al.” truncation was used after the first 30 authors, virtually all authors would be named in the citations as the 95% confidence interval, included an upper bound of 100.0% of the authors.

### Methods

The top 10 journals (as of 2019) in the disciplines of Plastic Surgery, Surgery, and Medicine were identified. Where possible the SCImago Institutions Ranking website was used. This website ranks journals based on their SCImago Journal Rank (SJR) indicator, which is a measure of journal impact, influence, or prestige. The SJR indicator expresses the mean number of weighted citations received in the selected year by the documents published in the journal in the three previous years. As Plastic Surgery is grouped under Surgery on the SCImago website, this website could not be used to determine the top Plastic Surgery journals. As such, a 2016 systematic review^15^ was used to help identify the top Plastic Surgery Journals. The top journals for Surgery and Medicine journals were identified on the SCImago website using the SJR number. After identifying the top journals, we reviewed the “Instructions for Authors” document for each journal to identify each journal’s criteria for using the “et al.” truncation. The Impact Factor and h-index for each of the selected journals were also found and presented (see Tables 1-3). Both measurements were found through the SCImago website or directly from the website of the respective journals. The Impact Factor measures the “importance of a journal by calculating the number of times selected articles are cited within a particular year.”^16^ The h-index, originally a measure used to describe how often an author was cited, is now assigned to journals.^16,17^ This measure is a reflection of the number of papers that have been cited (H), and how often, when compared to those that have not been cited (or cited as much). Publishing in a journal with a high h-index increases chances of being cited.^17^

**Table 3.** The Top 10 Surgery Journals, Rankings, and Use of “et al.” in Work Cited Page.

Journal	IF	h-index	SJF	Citation Rules
*JAMA Surgery*	13.625	170	3.757	7 or more, list the first 3 authors followed by “et al.”
*Annals of Surgery*	9.203	298	3.762	4 or more, list first 3 authors followed by “et al.”
*JNNP*	8.234	200	3.265	5 or more authors list the first 3 authors followed by “et al.”^a^
*JHLT*	7.865	130	3.945	7 or more, list the first 3 authors followed by “et al.”
Am. J. Surg. Pathol.	6.155	203	2.734	4 or more, list the first 3 authors followed by “et al.”
*BJS*	5.676	195	2.307	7 or more, list the first 6 authors followed by “et al.”
*JACS*	4.590	168	2.095	5 or more, list the first 3 authors followed by “et al.”
*JBJS*	4.578	249	2.482	Does not use “et al.” —cites all authors
*JNIS*	4.46	49	2.091	7 or more, list the first 3 authors followed by “et al.”
*Bone and Joint Journal*	4.306	172	2.375	7 or more, list the first 3 authors followed by “et al.”

Note: Journal guidelines were reviewed in February 2021.

^a^ Was not clearly stated—information found through reviews of recently published content.

Abbreviations: Am. J. Surg. Pathol., American Journal of Surgical Pathology; BJS, British Journal of Surgery; IF, Impact Factor; JAMA; Journal of the American Medical Association; JNNP, Journal of Neurology, Neurosurgery & Psychiatry; JHLT, Journal of Heart and Lung Transplant; JACS, Journal of American College of Surgeons; JBJS; Journal of Bone and Joint Surgery; JNIS, Journal of NeuroInterventional Surgery.

To address the secondary objective of this paper, one article was selected from each of the 30 identified journals. Articles were selected based on their date of publication (April or May 2020, whichever was more recent) and their “type” of publication, only Systematic Reviews or Original Articles were selected (ie Commentaries, Expert Opinion or Briefs were not selected). The first or second listed article that met the aforementioned criteria were selected; every effort was made to have a balance between systematic reviews and research articles, as well as long and short reference lists. After the article was selected, the reference section was copied into Microsoft Word; this will be referred to as “Original Reference List” for the remainder of the article. Using the Original Reference List, a new hypothetical “Extended Reference List” was created by replacing all “et al.” truncations with the names of all authors listed in the byline of the original publications. The Extended Reference List used the new “rule”: “if there are 31 or more authors, list the first 30 authors, followed by “et al.”. To determine the extra print “burden” posed by the Extended Reference List, the number of pages dedicated to the reference section in the Original and Extended Lists was compared. We then determined how many authors would be listed as opposed to missed if the Extended Reference List was used.

### Results

The 30 selected journals, by discipline, their rankings, and their “rules” for the use of “et al.” can be found in Tables 2 to 4. For all Plastic Surgery journals, if there were seven or more authors on an article, the “et al.” truncation was used after the first three authors; this “rule” is in accordance with the AMA citation style. In the Surgery and Medical journals, the “rule” was inconsistent. One journal in Surgery (*Journal of Bone and Joint Surgery – Series A*) and one Medical journal (*Physiological Reviews*) did not subscribe to the use of “et al.” and instead listed all authors.

**Table 2.** The Top 10 Plastic Surgery Journals, Rankings, and Use of “et al.” in Work Cited Page.

Journal	IF	h-index	SJR	Citation Rules
*PRS*	4.209	171	1.916	7 or more, list the first 3 authors followed by “et al.”
*ASJ*	3.799	53	1.587	7 or more, list the first 3 authors followed by “et al.”
*F&A*	3.787	NA	NA	7 or more, list the first 3 authors followed by “et al.”
*Head & Neck*	2.538	120	1.16	7 or more, list the first 3 authors followed by “et al.”
*JPRAS*	2.39	89	0.991	7 or more, list the first 3 authors followed by “et al.”
*Otolaryngol. Head Neck Surg*	2.31	116	1.18	7 or more, list the first 3 authors followed by “et al.”
*JHS Euro*	2.29	75	0.938	7 or more, list the first 3 authors followed by “et al.”
*JHS America*	2.09	111	1.258	7 or more, list the first 3 authors followed by “et al.”
*Int. J. Oral Maxillofac. Surg*	2.07	96	1.02	7 or more, list the first 3 authors followed by “et al.”
*Burns*	2.07	96	0.909	7 or more, list the first 3 authors followed by “et al.”

Note: Journal guidelines were reviewed in February 2021.

Abbreviations: ASJ, Aesthetic Surger*y* Journal; F&A, Facial Plastic Surgery and Aesthetic Medicine; IF, Impact Factor; Int. J. Oral Maxillofac. Surg, International Journal of Oral and Maxillofacial Surgery; JPRAS, Journal of Plastic Reconstructive and Aesthetic Surgery; JHS Euro, Journal of Hand Surgery European Volume; JHS American, Journal of Hand Surgery American Volume; NA, Not Applicable; Otolaryngol. Head Neck Surg, otolaryngology head and neck surgery; PRS, Plastic and Reconstructive Surgery.

**Table 4.** The Top 10 Medical Journals, Rankings, and Use of “et al.” in Work Cited Page.

Journal	IF	h-index	SJR	Citation Rules
*NEJM*	74.699	987	18.291	7 or more, list the first 3 authors followed by “et al.”
*The Lancet*	60.392	747	14.554	7 or more, list the first 3 authors followed by “et al.”
*Nat Rev Immunol*	40.358	371	20.611	6 or more, list first author followed by “et al.”
*Nat Medicine*	36.130	524	15.812	6 or more list first author followed by “et al.”
*J Clin Oncol*	32.956	525	10.054	4 or more, list the first 3 authors followed “et al.”
*Nat Rev Disease Pathol*	30.39	71	12.268	6 or more list first author followed by “et al.”
*Physiol Reviews*	25.588	330	10.153	Does not use “et al.”– cites all authors
*Acc Chem Re*	21.661	376	8.693	Does not use “et al.”
*Lancet Global Health*	21.597	NA	8.055	7 or more, list the first 3 authors followed by “et al.”
*Annu Rev Pathol*	16.75	112	8.508	7 or more, list the first 5 authors followed by “et al.”

Note: Journal guidelines were reviewed in February 2021.

^a^ Was not clearly stated—information found through reviews of recently published content.

Abbreviations: Acc Chem Re, Accounts of Chemical Research; Annu Rev Pathol, Annual Review of Pathology: Mechanisms of Disease; J Clin Oncol, Journal of Clinical Oncology; Nat Rev Disease Pathol, Nature Reviews Disease Pathology; NA, Not Available; NEJM; New England Journal of Medicine; Nat Rev Immunol, Nature Reviews Immunology; Nat Medicine, Nature Medicine.

A total of 30 articles, one article from each journal in the 3 categories (eg Plastic Surgery, Surgery, Medicine), were selected and reviewed. In the *Plastic Surgery* category (Table 5), two articles required one additional page to accommodate the Extended Reference List. In one of these articles (published in *Head & Neck*), the addition of one page allowed nearly 300 authors to be given credit for their work as opposed to using “et al.”. In three (30%) of the Plastic Surgery articles, the use of the “et al.” truncation was not seen. This was not due to the journal's policies on citation, but simply because none of the referenced articles had an author list longer than 7 authors. In the other five (50%) Plastic Surgery articles, no additional pages were needed to accommodate the Extended Reference List. Despite not needing any additional space, the use of the new rule (list 30 authors followed by “et al.”) resulted in 10 to 60 more authors being cited, and thus recognized.

**Table 5.** Original Versus Extended Reference Information for Plastic Surgery Journals.

Journal	Original Reference List			Extended Reference List	
	Page Count	Total References	# of References using “et al.”	Page Count	# of additional authors listed
*PRS*	3	35	5	3	32
*ASJ*	2	19	1	2	10
*F&A*	2	14	0	2	0
*Head & Neck*	3	32	28	4	297
*JPRAS*	3	22	7	3	60
*Otolaryngol. Head Neck Surg*	3	37	5	3	33
*JHS Euro*	2	15	0	2	0
*JHS American*	4	26	5	4	50
*Int. J. Oral Maxillofac. Surg*	4	34	0	4	0
*Burns*	5	52	6	6	44

Note: Bolded, additional pages were needed for Extended Reference List.

Abbreviations: ASJ: Aesthetic Surgery Journal; F&A, Facial Plastic Surgery and Aesthetic Medicine; Int. J. Oral Maxillofac. Surg, International Journal of Oral and Maxillofacial Surgery; JPRAS, Journal of Plastic Reconstructive and Aesthetic Surgery; JHS Euro, Journal of Hand Surgery European Volume; JHS American, Journal of Hand Surgery American Volume; Otolaryngol. Head Neck Surg, otolaryngology head and neck surgery; PRS; Pla*s*tic and Reconstructive Surgery; #, Number.

When looking at the 10 articles from the *Surgery* category (Table 6), three articles (30%) required one additional page to accommodate the Extended Reference List. In one article, published in the *British Journal of Surgery*, the addition of one page allowed for 239 more authors to be recognized for their work. Seven articles (70%) did not require any additional pages to accommodate the new rule. Even without adding additional pages, 56 to 353 additional authors were cited for their work. Lastly, in the *Medicine* category (Table 7), 5 (50%) of the articles required additional pages to accommodate the Extended Reference List. The article published in *Nature Reviews Disease Pathology,* required the most additional pages (7 pages), however, through using the Extended Reference List, 1789 more authors were acknowledged.

**Table 6.** Original Versus Extended Reference Information for General Surgery Journals.

Journal	Original Reference List			Extended Reference List	
	Page Count	Total References	# of References using “et al.”	Page Count	# of additional authors listed
*JAMA Surgery*	3	20	9	3	56
*Annals of Surgery*	3	32	20	3	70
*JNNP*	8	59	55	8	353
*JHLT*	3	22	12	4	147
*Am. J. Surg. Pathol.*	3	30	24	3	171
*BJS*	8	111	47	9	239
*JACS*	3	23	16	4	88
*JBJS*	2	23	0	2	0
*JNIS*	3	23	19	3	125
*Bone and Joint Journal*	4	32	10	4	69

Note: Bolded, additional pages needed for Extended Reference List.

Abbreviations: American Journal of Surgical Pathology, Am. J. Surg. Pathol; BJS, British Journal of Surgery; JAMA; Journal of the American Medical Association; JNNP, Journal of Neurology, Neurosurgery & Psychiatry; JHLT, *J*ournal of Heart and Lung Transplant; JACS, Journal of American College of Surgeons; JBJS; Journal of Bone and Joint Surgery; JNIS, Journal of NeuroInterventional Surgery; #, Number.

**Table 7.** Original Versus Extended Reference Information for Medicine Journals.

Journal	Original Reference List			Extended Reference List	
	Page Count	Total References	# of References using “et al.”	Page Count	# of additional authors listed
*NEJM*	3	23	10	3	114
*Lancet*	6	57	18	6	204
*Nat Rev Immunol*	10	126	66	12	732
*Nat Medicine*	6	63	39	9	795
*J Clin Oncol*	3	32	28	4	352
*Nat Rev Disease Pathol*	53	280	166	60	1789
*Physiological Reviews*	40	482	0	40	0
*Acc Chem Res*	5	25	0	5	0
*Lancet Global Health*	8	76	47	10	683
*Annu Rev Pathol*	5	51	9	5	43

Note: Bolded, additional pages needed for Extended Reference List.

Abbreviations: Acc Chem Re, Accounts of Chemical *R*esearch; Annu Rev Pathol, Annual Review of Pathology: Mechanisms of Disease; NEJM; New England Journal of Medicine; Nat Rev Immunol, Nature Reviews Immunology; Nat Medicine, Nature Medicine; Nat Rev Disease Pathol, Nature Reviews Disease Pathology.; #, Number.

## Discussion

The present article addresses an important issue; the recognition of all investigators participating in clinical research. The citation format presently used by most journals does not do justice to most authors in the reference list at the end of an article. Admittedly, if one wants to find the complete authorship list of an article, all one has to do is search, find, and read the original article. This however adds an additional burden on the reader, and some consider it an insult to not be cited within a reference section just as in our scenario. The people frequently not cited are the supervisors, or senior authors, who may have contributed much more to the research project in terms of scientific ideas and relevance of the project than the first author who may be a medical student or resident. Senior surgeons, who act as mentors usually do not want to be seen as competing with their mentees as these young learners’ transition from a supervised environment to an independent research or clinical practice. Although we support the “mentor” “mentee” relationship and have the “mentees” listed as the first authors, this altruistic approach should not penalize the senior mentors by not citing their names in the reference lists, particularly if they are listed last as the senior author. This problem can easily be avoided by including all authors of a published piece in the reference section at the end of an article. Our study found that the “et al.” truncation kicks in variously in journals across genres of Plastic Surgery, Surgery, and Medicine.

Of the 30 journals included in this review, all but two (one in Surgery and one in Medicine) utilized the “et al.” truncation and therefore do not recognize all authors. As we review the results from Table 5, we see that in Plastic and Reconstructive Surgery Journal (PRS), the flagship journal of our specialty, not a single extra page was added, yet 32 additional authors were cited and thus recognized if the Extended Reference List was used. We believe that all journals should consider the following rule, *list all authors up to 30, followed by “et al.”.* If all journals made the effort to include a maximum of 30 authors before the “et al. ” appears, almost 100% of authors would be recognized. This confirms earlier work by Goldsmith and colleagues,^14^ when the “et al.” truncation is introduced after the author number exceeded 30 there was an almost 100% chance of all authors being included. The adoption of this rule rarely results in the need for addition space (measured in pages), especially within Plastic Surgery. Out of the 10 articles reviewed within the category of Plastic Surgery, one required more space to accommodate the “Extended Reference List”. The benefit of this one additional page, however, was huge as an additional 39 authors were acknowledged. The largest increase in pages dedicated to the Extended Reference list was found to be 3 pages; however, the addition of these pages resulted in nearly 800 additional authors being recognized. This means that, in all likelihood, all contributors, including senior authors, methodologists, biostatisticians, and health economists would be included in the referenced citations

Bettering the issue of the “et al.” truncation should be tackled by all individuals involved in clinical research, from design to publication. As the “final decision” as to whether a manuscript is published, or an abstract is accepted to a conference, is in the hands of journal editors and conference organizers, the adoption of a new way of citing published work begins with them. While some journals use the “et al.” truncation in an effort to save space, those who publish either both online and in print, or only online, should consider applying the “Extended Reference List” when possible. This would mean properly recognizing all authors by listing all authors up to 30, followed, only then, by the “et al.” truncation. The same principle should also apply to meetings/conferences. If such policies are changed, this will allow researchers to properly recognize other authors within their own work, without the fear of having a submitted manuscript or abstract rejected. Once policies are changed, this will allow the doers of research to make changes at their level. For example, surgeons who supervise young researchers (eg medical students, residents, or junior faculty) could then openly encourage the citing of all authors, or at least the use of “et al.” only if 31 authors or more are cited in an article. This could be adopted not only in manuscript submissions but also in conference abstracts, Master’s theses, Doctoral dissertations, and the curriculum vitae (CV) of the mentee. Through the adoption of the new citation rule, it is almost certain that the complete author list will be recognized.

In this article, we attempted to show that the “et al.” truncation in the reference list of Plastic Surgery articles (as well as other journals) is an anachronism that needs to be changed and thus gives recognition to all authors by citing their names. The study, of course, has some limitations. First, the number of articles we reviewed was small. Perhaps, a larger “page burden” may have resulted if more articles were reviewed, however, our guess is that the conclusions as a whole would not substantially change. The current study supports an earlier report that examined the nonrecognition of statisticians in published literature.^14^ Ultimately, we are suggesting that it is not up to journals or meeting organizers to decide who gets recognition for their work; all authors should be acknowledged. Based on the evidence provided, we strongly encourage the journal editors and publishers to revise the existing citation truncation and list all authors up to 30 before the “et al.” is used.
